# Supplementary material for: A plasma membrane template for macropinocytic cups
Source: eLife. 2016 Dec 13;5:e20085. doi: 10.7554/eLife.20085 (PMC5154761; doi:10.7554/eLife.20085)
Supplement: Supplementary file 1. — DOI: http://dx.doi.org/10.7554/eLife.20085.029 [file elife-20085-supp1.pdf]

Introduction

This document is a user manual for the MATLAB script track_membrane. The purpose of track_membrane is to quantify the fluorescence signal along the cell membrane.

The program takes a pragmatic approach, relying much on human input. Rough cell outlines are drawn, checked and corrected manually. The program only makes small adjustments to find the maximal fluorescence signal along the cell contour. If a more unbiased approach is preferred, it is also possible to identify the membrane contour automatically, based on thresholding.

Quantifying fluorescence intensity levels on the cell membrane is particularly challenging, because the membrane defines the edge of a compartment. Fluorescence levels outside of the cell are naught and as a result only small errors in the correct identification of the membrane can lead to sharp decreases in measured fluorescence. It also complicates co-localisation studies as the co-compartmentalisation of both fluorophores in the cell can easily be mistaken for co-localisation if the membrane contour is not determined with sufficient care.

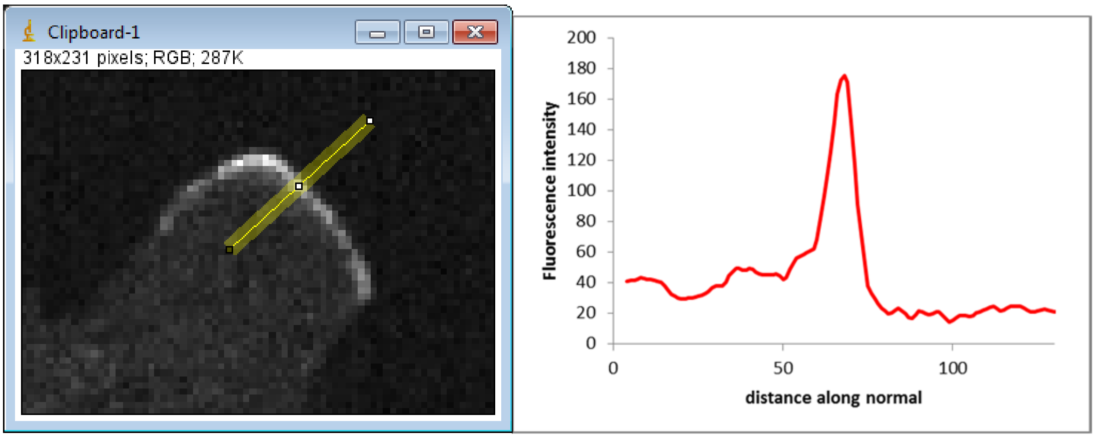

Membrane fluorescence intensity levels in track_membrane are determined by first drawing a normal on the cell contour and then determining the maximum fluorescence level along this normal. If there is no enrichment of fluorescence on the membrane, then the identified signal is equal to that of the cytosol background, since the normal extends somewhat into the cytosol.

Output can be written to the MATLAB workspace, exported to comma-separated files and also some basic graphs can be created by the program itself.

Please report bugs or suggestions for improvement,

Douwe Veltman
d.m.veltman@rug.nl

Contents

Introduction 1

Workflow 2

Prepare the images using ImageJ. 2

Start the track_membrane script.. 2

Open the image file.. 3

Draw the cell contour 3
Display the output data 6
Loading / saving contours 7
Positioning the starting point of the membrane contour 7
Normalisation. modes – local and global cytosol measurement........ 8
Exclusion regions – removing unwanted points 9
Automated outlining........ 11
Selecting output graphs 12
Identifying points of interest 13
Key list........ 14

General workflow

Prepare the images using ImageJ.

track_membrane is only able to open common file formats, such as .tif, .bmp and .jpg, whereas images from the microscope are usually in .lsm (Zeiss), .nd2 (Nikon) or .lei (Leica) format. These images can be imported in ImageJ with the help of the LOCI plugin. This plugin that is supported by the Open Microscopy Environment, allows a multitude of proprietary image formats to be read and is free to install (https://www.openmicroscopy.org/site/support/bio-formats5.1/users/imagej/).

Prepare imported images either as single channel (i.e. GFP only) or dual channel (i.e. GFP and RFP). ImageJ calls multi-channel images "composite images". Images can consist of a single time point or a time-lapse of multiple time points. ImageJ calls the latter an "image stack". Both 8-bit and 16-bit images are supported by track_membrane. Crop the images as far as possible to reduce memory usage and then save them as a .tif file.

Start the track_membrane script.

Whenever a command is typed into the MATLAB command window, MATLAB checks for the presence of a file with such a name in a number of folders. This collection of folders is called the "path". You can see which folders are checked my MATLAB by typing:

```
path
```

in the command window. Typically, the top entry would be something like "C:/Windows/Users/S12345678/MATLAB/". Copy the track_membrane script to this folder so that MATLAB can find it. Alternatively, you can add the folder containing the track_membrane script to the path by typing:

```
path (path, 'C:/folder/that/contains/track_membrane/')
```

When the script is in the MATLAB path, start it up by simply typing the script name in the command window:

```
track_membrane
```

Open the image file

After starting the script you will be presented with a black, empty window. The number of buttons and sliders are kept to a minimum. All interaction is done via the keyboard. Press "**o**" to open an image. You the window should then look something like:

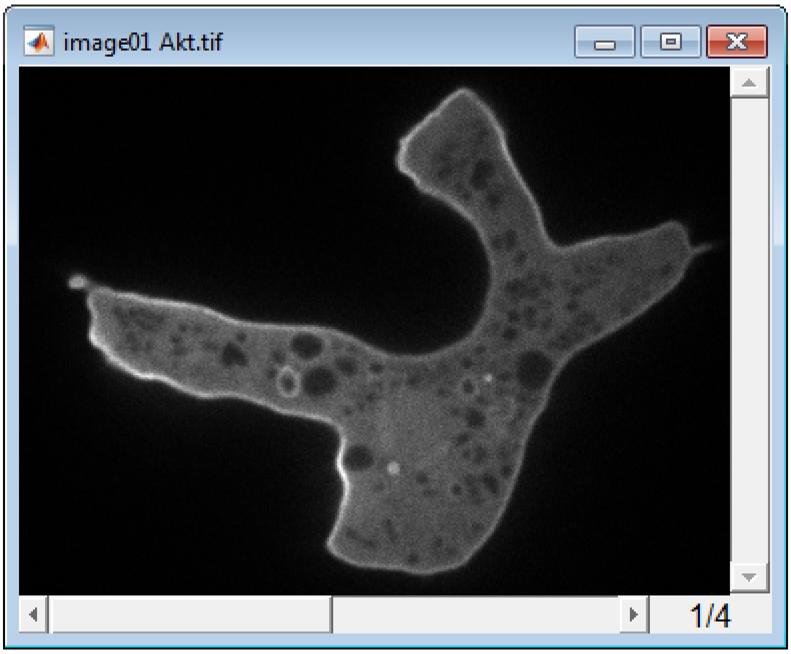

Numbers on the bottom-right indicate that this is frame 1 out of 4 frames in total. You can drag the slider at the bottom to go through all the images of the stack or use the left and right cursor keys.

The slider on the right is used for thresholding. More about that later.

You can use the scroll wheel of the mouse to zoom in and out of the image. Once you are zoomed in, you can hold the right mouse button for panning.

Draw the cell contour

Press the "**a**" key to add a membrane contour. The mouse pointer will change into a cross-hair. Keep the left mouse button pressed and draw the contour around the cell. The image should now look something like this:

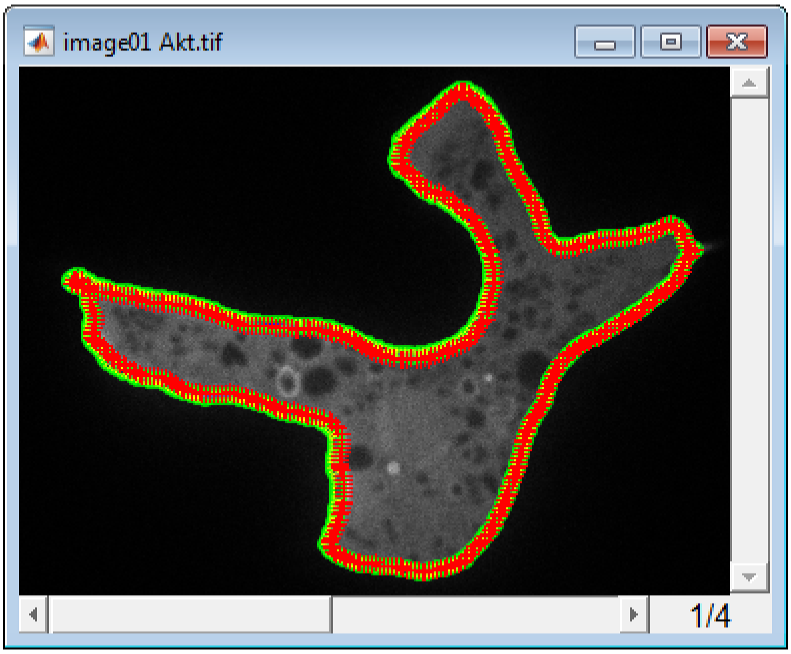

If you have made a mistake and would like to start again, press "**d**" to remove the contour.

Zoom in and pan around to see if the drawn contour follows the cell outline correctly. If you have drawn the contour a bit too wide and would like to trim it a bit more tightly, press "**t**" and draw the area that should be trimmed from the current contour like this:

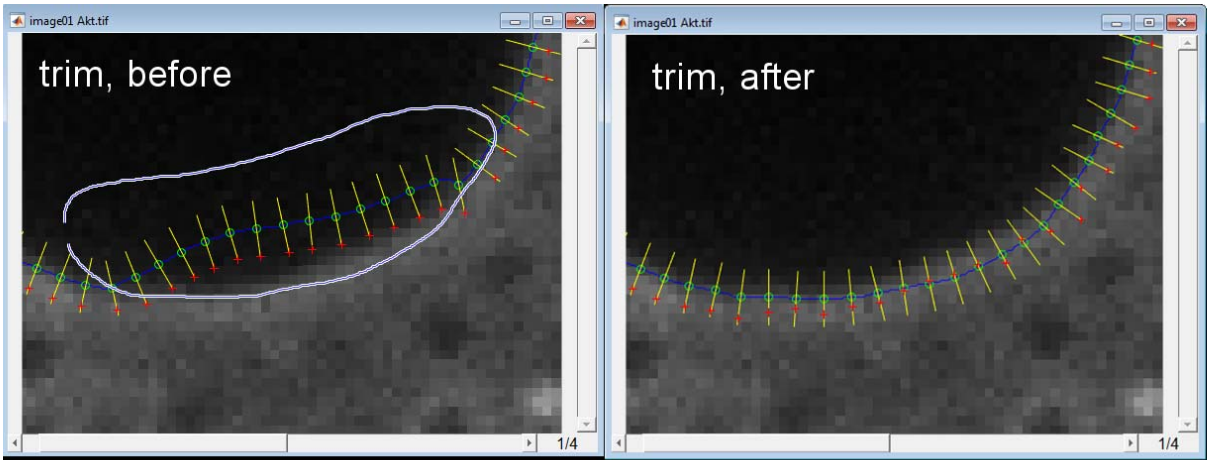

Vice versa, if you have drawn the contour a bit too narrow and would like to include more cell area, hit "**a**" again and draw the region to be added to the current contour like this:

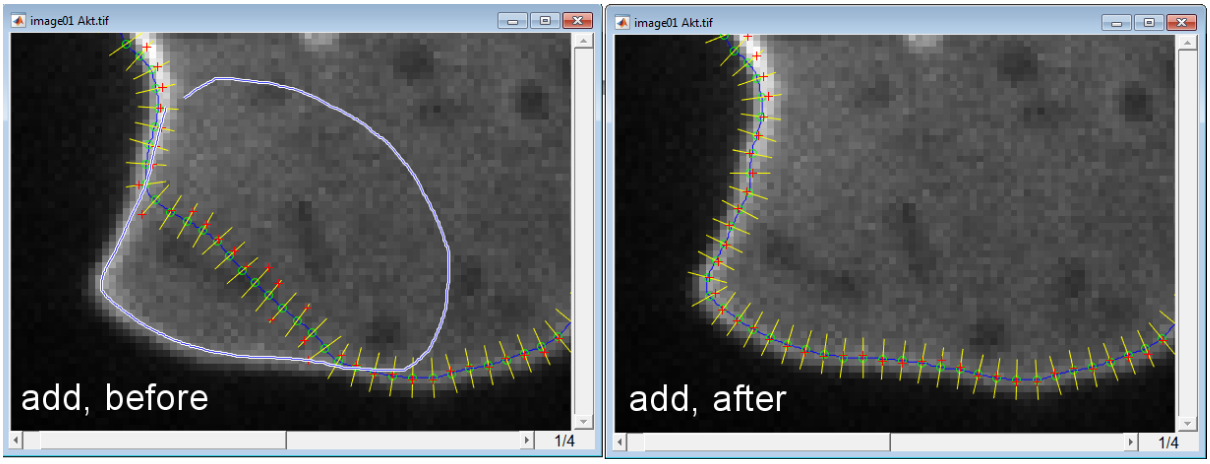

You can see how MATLAB tries to correctly track the cell membrane

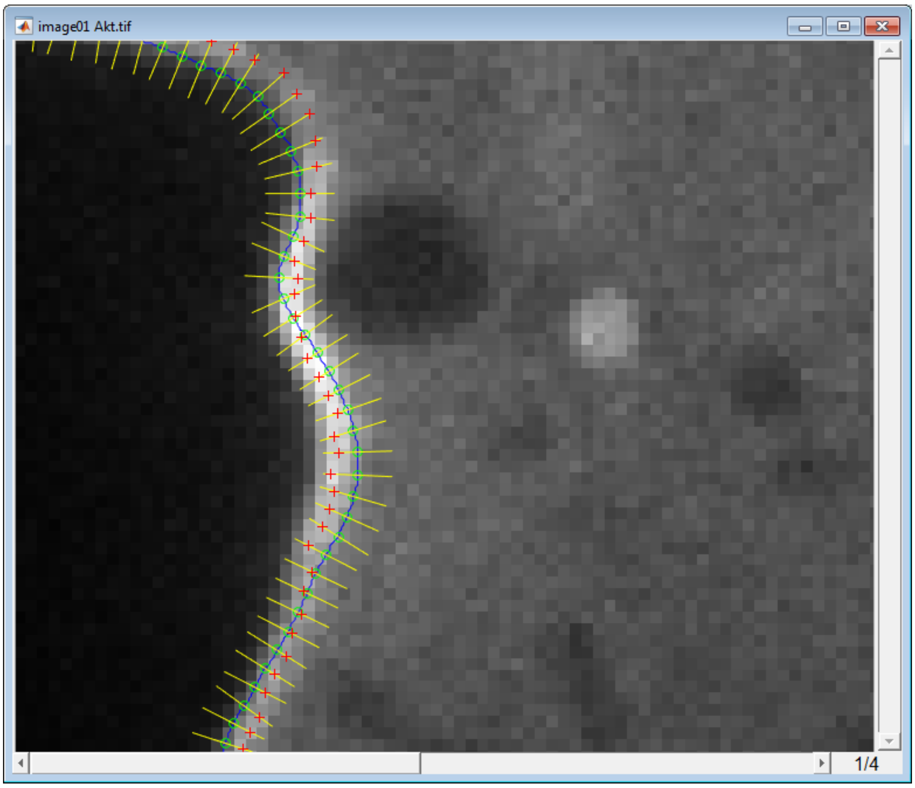

The blue line indicates the hand-drawn contour.

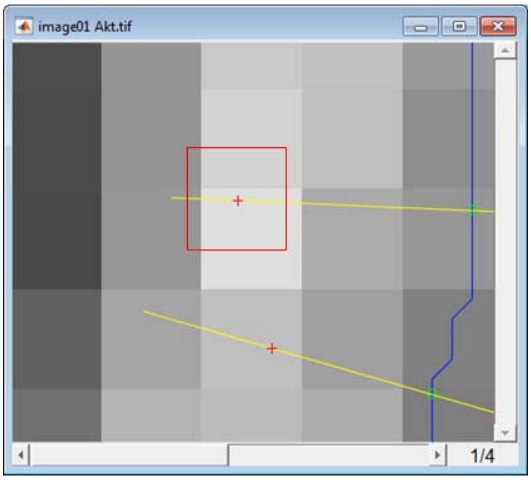

MATLAB draws equidistant points along the contour (green circles).

MATLAB draws a normal (= a perpendicular line) to the contour through each equidistant point (yellow line).

MATLAB scans along the normal and finds the point of maximal fluorescence intensity (red +)

For this scanning, it uses interpolation. Thus in the picture on the right, the pixel intensity measured at the point indicated by the + mark is not equal to the intensity of the bright pixel that it resides on, but it is the weighted average of the pixel values that are enclosed by the 1-pixel-sized box indicated in red.

Once you are happy with the drawn membrane contour, it is time to indicate the cytosol and background regions, so that MATLAB can calculate the membrane fluorescence intensity relative to the cytosol. This is analogous to the drawing of the membrane contour. Press "**alt-a**" and draw a region around a vesicle-free cytosol area (difficult to find in the example image) like this:

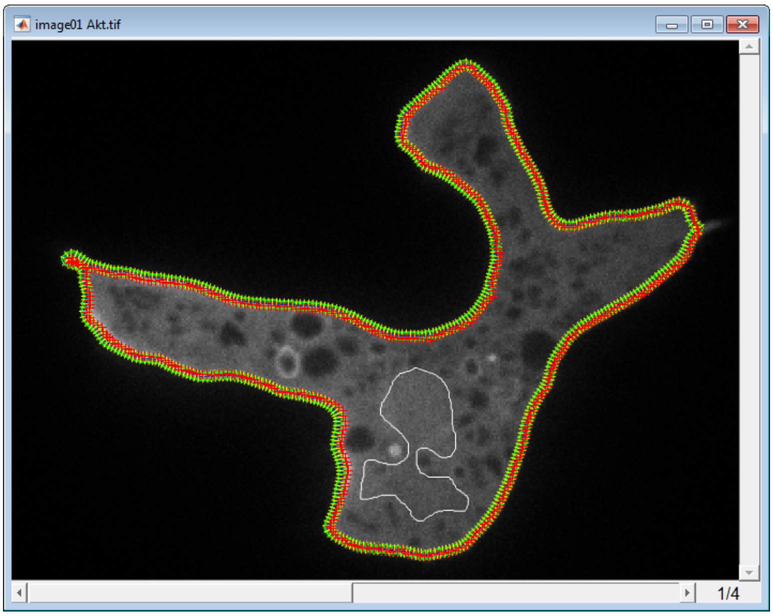

MATLAB will measure the cytosol fluorescence by taking the mean fluorescence of the area indicated by the white contour.

Now do the same thing for the background fluorescence. This is the fluorescence intensity that is measured in an area outside of the cell. Draw the background region by pressing "**control-a**" and draw an area like this:

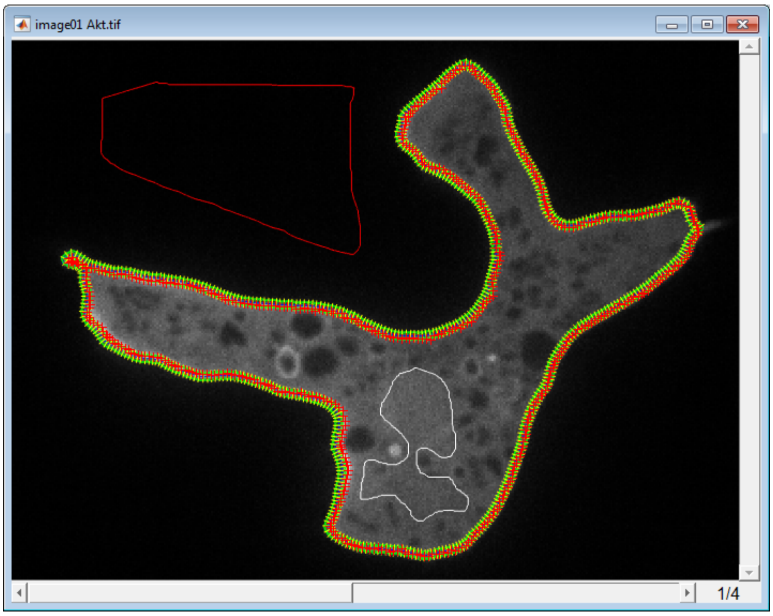

MATLAB will measure the background fluorescence by taking the mean fluorescence of the area indicated by the red contour.

Display the output data

MATLAB now has all the information it needs to plot the membrane fluorescence relative to the cytosol. This is calculated as follows:

(membrane – background) / (cytosol – background)

Press "**p**" to draw a plot of the fluorescence intensity along the membrane contour:

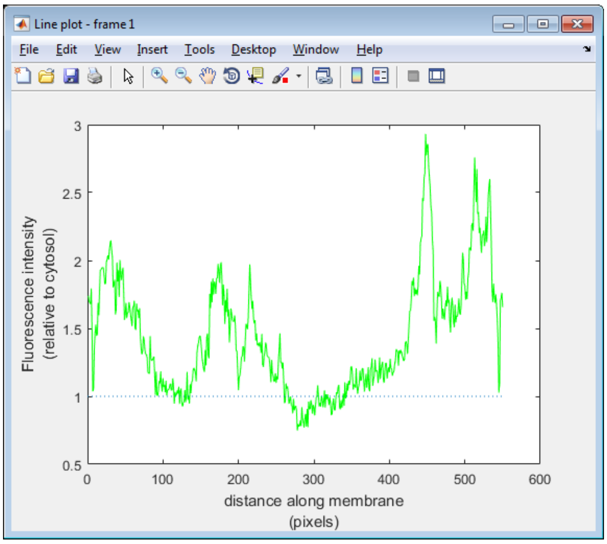

Fluorescence intensity is plotted in green. The horizontal dotted line indicates a relative fluorescence intensity of 1. In principle, the measured fluorescence intensity at the membrane should not be lower than 1 (i.e. not lower than the value of the cytosol).

Loading / saving contours

Drawing contours can be very time consuming. Save your work by pressing "**s**". A MATLAB data file containing all the contours will be written to disk. By default, the file will be saved in the same directory as that of the image and with the same name, but with the extension ".m". Alternatively press "**shift-s**" allows you to choose a folder and file name.

In the same way, saved data can be loaded by pressing "**l**". By default MATLAB will look in the same directory as the opened image file and look for a file with the same name as the opened image file, but with the extension ".m". Alternatively press "**shift-l**" to choose a different contour file.

Positioning the starting point of the membrane contour

By default, MATLAB starts the cell contour at the leftmost point (contour runs clock-wise). The starting point of the contour is indicated by a diamond. The end of the contour is indicated by a square (see image below. Middle panel is zoomed into the region indicated by the arrow)

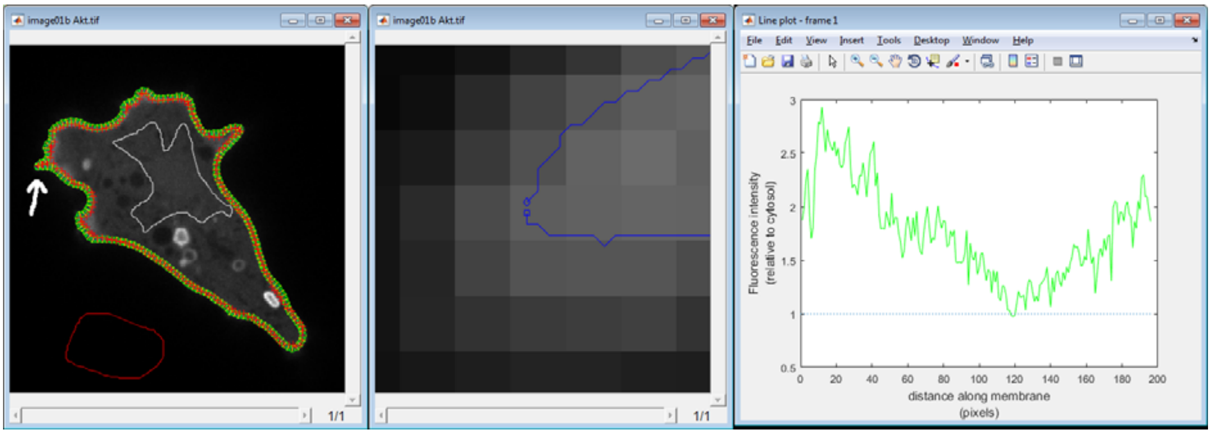

In most cases, it is preferred to have the front of the cell in the middle of the cell contour, but in the above example, this is not the case. The starting point of the contour can be set by placing a so-called "magnet point". Press "**m**" to place a magnet point. Select a point close to the rear of the cell:

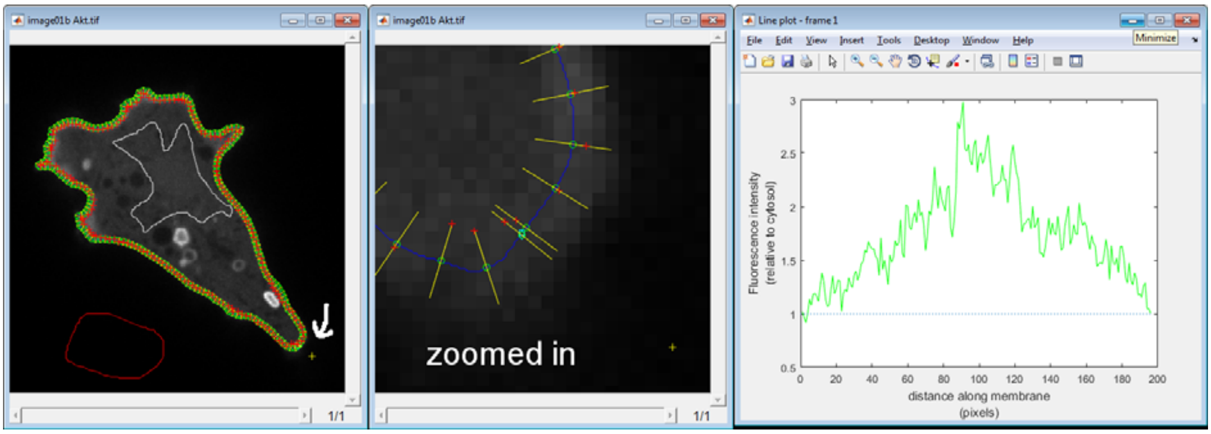

The arrow points to the magnet that was drawn. The start and stop are now positioned at the back. The colour of the start and stop point has changed to cyan to indicate that their position is determined by a magnet. The membrane fluorescence intensity plot in the right panel now shows the back of the cell at the edges of the plot and the front of the cell at the centre of the plot.

In an image stack with multiple frames, the magnet only needs to be set for the first frame. MATLAB will position the start of the contour in the current frame at the position closest to the start of the contour in the previous frame. If this is not desired, this position can be overridden by placing a new magnet. If you change the position of the magnet in an image stack, you may need to press "**control-u**" to update the contours of the stack so that change in the position is recalculated for all the sequential frames.

Normalisation. modes – local and global cytosol measurement

MATLAB normalises the membrane fluorescence relative to the fluorescence intensity of the cytosol. This makes it fairer to compare fluorescence intensities from different cells, which may show somewhat higher or lower expression levels. However, in some cases the normalisation using a single cytosolic fluorescence value is not sufficient. This can be the case when the fluorescence intensity of the cytosol is not homogeneous. For example, Dictyostelium pseudopods are vesicle-free zones. As a result, they contain more cytosol and appear brighter. Vice versa, uneven illumination can lead to cell areas that are dimmer, such as in the example below:

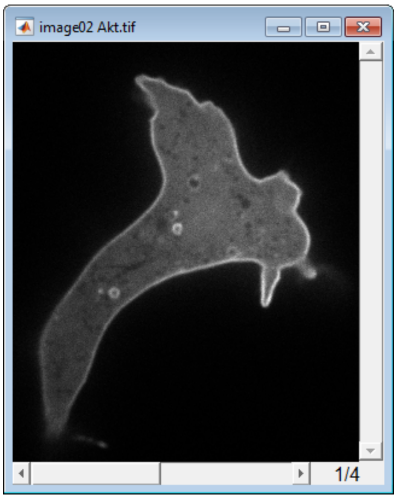

In this case, the uneven illumination causes the tail of the cell to be less bright. This can be corrected by measuring cytosol values locally. Pressing "**c**" switches between global (default) and local cytosol measurement. In local cytosol mode, MATLAB draws an inner contour at a fixed distance from the hand-drawn cell contour. It then measures the fluorescence intensity of the cytosol of each point

along this inner contour. For each point of maximal fluorescence on the membrane contour it finds the closest point on the inner contour and it uses this value to normalise the membrane fluorescence intensity:

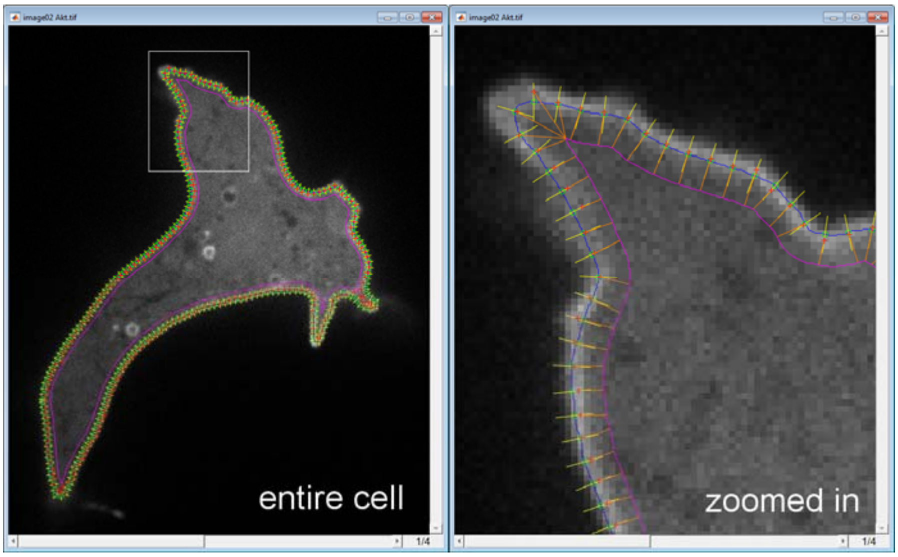

The orange lines connect the points of maximal fluorescence with their paired cytosol point. Their visibility can be toggled by pressing "**control-v**".

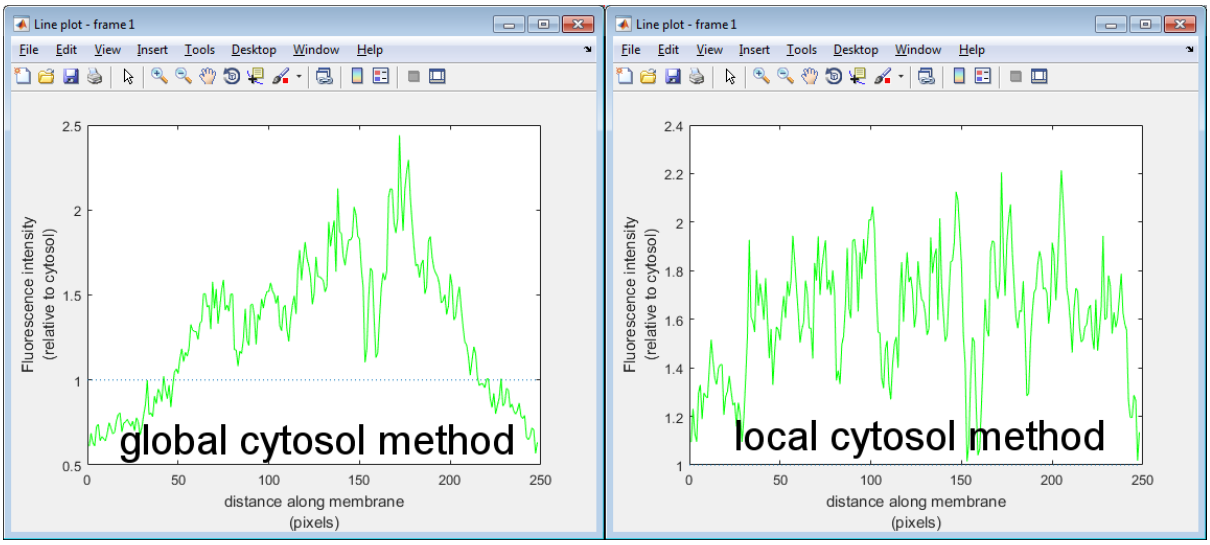

The difference in membrane fluorescence intensity measurements is clear. The global cytosol measurement method indicates that the membrane fluorescence intensity in the front of the cell is almost 4-fold higher than in the back, whereas with local cytosol correction, the membrane fluorescence intensity relative to the cytosol is rather homogeneous around the entire cell circumference.

Exclusion regions – removing unwanted points

Life is not perfect. You will find many regions where the fluorescence intensity of the membrane cannot be accurately determined. This can be either due to limitations of the microscope, when a cell is contacting another cell, when cellular debris obfuscate parts of the image, when part of the cell is out of focus or when intracellular vesicles come close to the cell membrane. These events can

lead to erratic measurements that must be excluded from the dataset. See for example the image below.

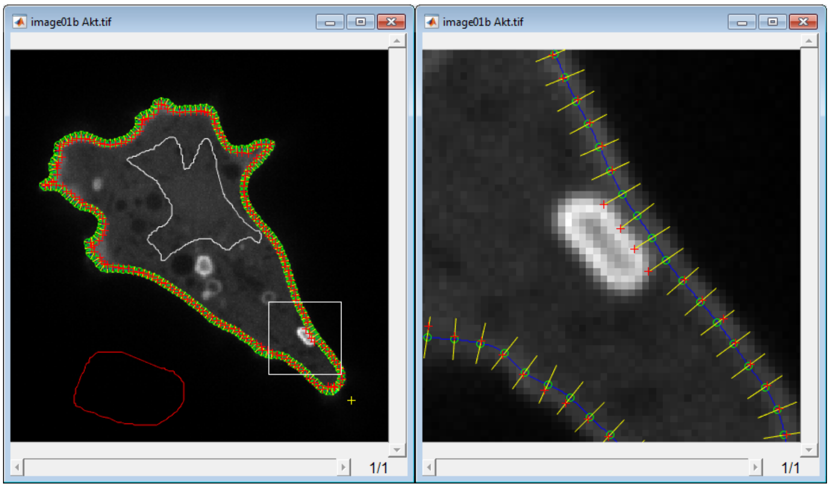

The point of maximum fluorescence is part of the vesicle that encloses the ingested bacterium, not part of the cell membrane. Exclusion regions can be drawn by pressing "**shift-a**". See image below. Exclusion regions are shaded in red.

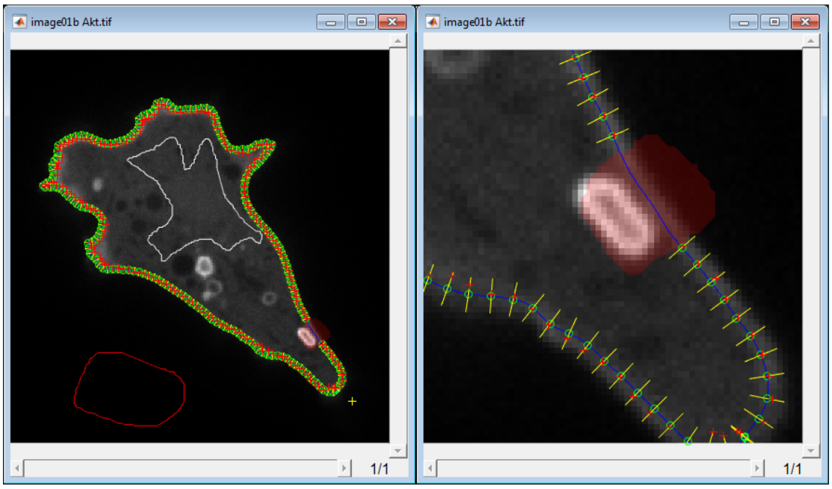

Multiple independent regions can be drawn for each frame. Points that lie within exclusion regions are not included in measurements. The left panel in the image below shows that the phagocytosed bacterium in the cell leads to an apparent sharp peak in membrane fluorescence. These points are excluded in the image on the right.

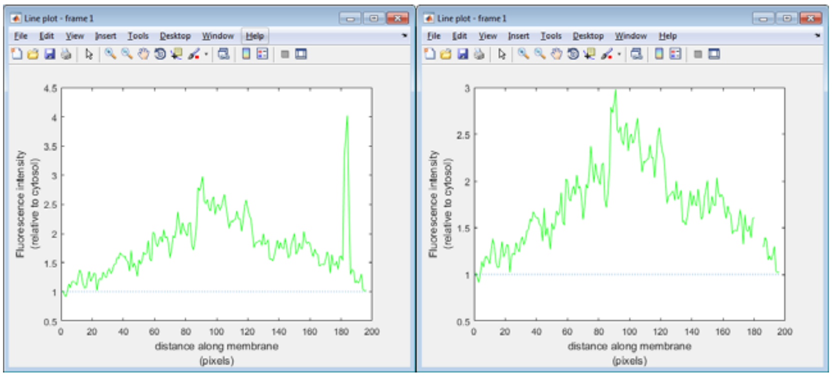

Use this function for good, not for evil! (Note that in this particular case, the problem could also be solved by reducing the length of the normals (using the "**[**" and "**]**" keys) or by drawing the membrane contour a bit wider so that the normals no longer reach the bacterium).

Automated outlining

Manual outlining is usually gives the best results, but can be tedious. MATLAB is able to automatically outline the cell using thresholding. Press "**f**" to enable the thresholding slider on the right hand of the screen. Drag the slider up and down to change the threshold. Pixels that are above threshold are indicated in semi-translucent white.

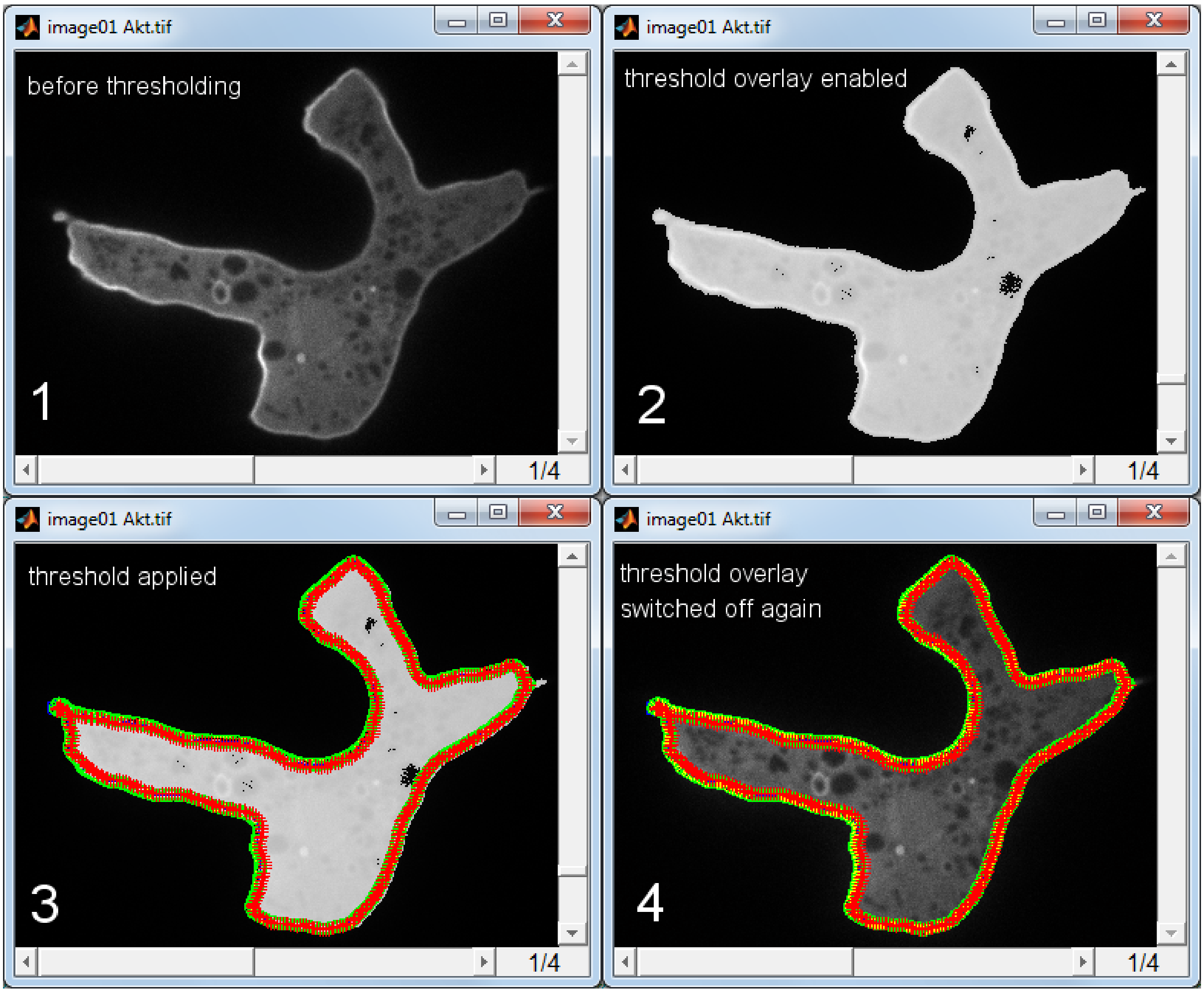

Apply the threshold by pressing "**enter**". MATLAB draws the cell outline based on the threshold. If this is too narrow or too wide, change the threshold value accordingly by dragging the slider and re-apply the threshold. This will replace the previous threshold. Check the threshold and if necessary, make some corrections by manually adding or trimming regions as explained previously. Press "**f**" again to toggle the threshold overlay off again. MATLAB remembers the threshold value, so it can be immediately applied to following frames by simply pressing "**enter**".

In an image stack, contours can be automatically copied from adjacent frames using the cursor keys, which can save a lot of time and increases consistency across frames:

control-→
Go to next frame and draw a copy the background region of the current frame.
alt-→

Go to next frame and draw a copy the cytosol region of the current frame.
shift-→
Go to next frame and automatically draw a membrane contour, based on the current threshold.

control-←
Go to previous frame and draw a copy the background region of the current frame.
alt-←
Go to previous frame and draw a copy the cytosol region of the current frame.
shift-←
Go to previous frame and automatically draw a membrane contour, based on the current threshold.

It should be noted that in an image stack the outline from the previous frame is always drawn as a dashed line. This line can serve as a guide for drawing the contour in the current frame and can also improve frame-to-frame consistency.

Selecting output graphs

Output and other options can be selected by pressing "**g**".

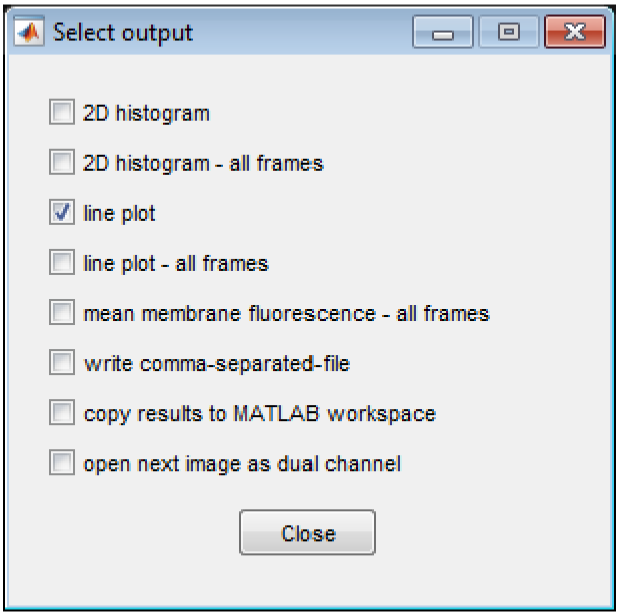

2D histogram
Requires a dual channel image. For all points in the current frame, the fluorescence intensity of channel 2 is plotted against the fluorescence intensity of channel 1.

2D histogram - all frames
As above, but now uses the data of all the frames in the stack

line plot
Plots the fluorescence intensity along the cell outline. Works for both single and dual channel images (this plot is checked by default)

line plot - all frames
For each frame, the identified membrane pixels are plotted along a vertical line and then all vertical lines of the frame are horizontally concatenated to yield an image like this:

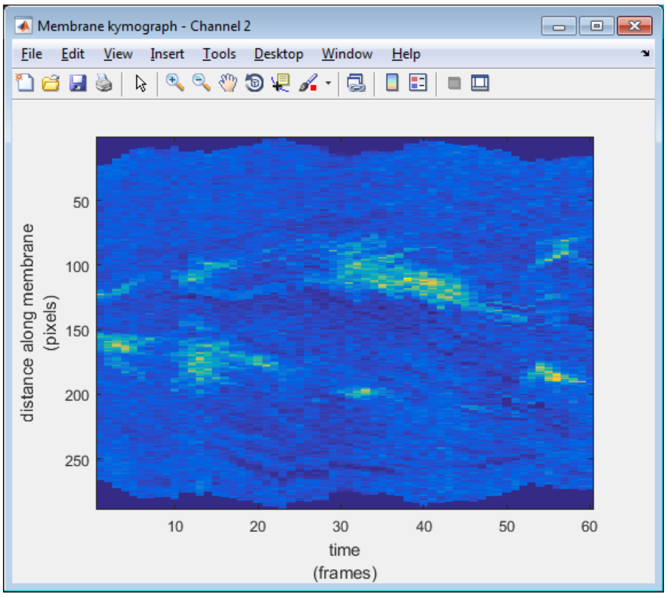

<u>mean membrane fluorescence - all frames</u>
The mean fluorescence intensity of all the pixels in the contour is plotted for each frame in the stack.

<u>write comma-separated file</u>
A comma separated file with the normalised membrane fluorescence is written to disk (a centred column vector for each frame. Thus, the same layout as the plot above). By default the file name and location is identical to that of the opened image file, but with the extension .csv. For dual channel images a separate file is written for each channel.

<u>copy results to MATLAB workspace</u>
For each channel the fluorescence intensity of the membrane, the cytosol, the background and also the calculated normalised fluorescence intensity is written as a matrix to the default MATLAB workspace:

```
channel1_background
channel1_cytosol
channel1_membrane
channel1_normalised
```

<u>open next image as dual channel</u>
When this box is ticked, the next image that is opened (by pressing "**o**") is assumed to be a dual channel image.

Identifying points of interest

The data can be further analysed by linking points in a 2D histogram back to the cell image. This can be useful for example to determine the identity of outliers and other extremities in the dataset.. Outline all the cells in a dual-channel stack. Press "i" to draw a 2D histogram and circle the data points of interest. These points will now be highlighted in the image using red asterisks (*).

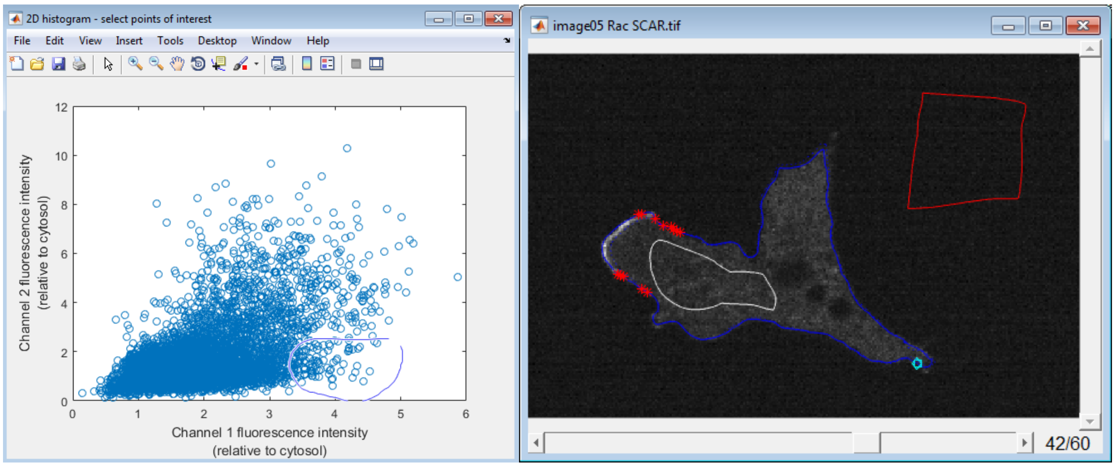

Points of interest are selected on the left and shown in the image on the right.

Key list

a

Manually draw the membrane contour. If a membrane contour already exists, then the new contour is added to the old contour. If the new contour does not overlap with the old contour, then it is disregarded.

control-a

Same as above, but now for the region that contains background signal. The colour of this contour is red.

alt-a

Same as above, but now for the region that contains cytosol signal. The colour of this contour is white.

shift-a

Manually draw an exclusion region. Membrane points that fall within this region will not be measured. It is possible to draw multiple different regions. Exclusion regions are shaded in red.

c

toggle between local and global cytosol measurement. The default measurement method is global.

d

Delete the membrane contour of the current frame.

control-d

Delete the background region of the current frame.

alt-d

Delete the cytosol region of the current frame

shift-d

Delete the exclusion regions of the current frame

f

Toggle threshold visibility on/off.

g

choose graphs and/or select whether the opened image is dual channel

i

Displays a 2D histogram of the data of the current frame and allows a region to be drawn around a group of points of interest. These points will then be highlighted in the image with a red asterisk.
shift-i
Same as above, but now using a 2D histogram of the data from all frames in the stack.
alt-i
Delete the points of interest.

k
Toggle between colour maps 'gray' and 'jet'. Default colour map is 'gray'. The 'jet' colour map allows better visibility of low-intensity pixels.

l
Load membrane contours that have previously been saved by pressing "s".
shift-l
Same as above, but now a file dialog appears that allows you to choose which membrane contour file you want to load.

m
Select a "magnet" point. The membrane contour will start at the point that is closest to the magnet. The magnet is depicted as a yellow "+".
alt-m
Delete the magnet point from the current frame.

o
Open a new image. Both single images and image stacks can be opened. By default image stacks are opened as single channel. Tick the dual channel box in the graphs dialog (by pressing g) if you want the image stack to be opened as dual channel.

p
Measures the intensity values of all identified points and generates the plots indicated in the graphs dialog box (can be opened by pressing g). If no background region or cytosol region have been selected then a normalised membrane signal cannot be calculated and no graphs will be drawn.

s
Save the membrane contours to file. The filename will be the same as the name of the image file, but with the extension .mat
shift-s
Same as above, but now a specific file name can be chosen.

t
Manually draw a region that will be trimmed from the current membrane contour.
control-t
Same as above, but now for the region that contains background signal.
alt-t
Same as above, but now for the region that contains cytosol signal.
shift-t
Same as above, but now for the exclusion regions.

u
Update the membrane contour.
shift-u

Update the all membrane contours of the stack. Updating may be required when for example a magnet is added or removed.

v
Toggles the visibility of the equidistant membrane points, normals and maximum intensity points
control-v
Toggles visibility of the connectors between the maximum intensity points and the paired cytosol point.
alt-v
Toggles visibility of the Points of Interest
shift-v
Toggles visibility of the contour of the previous frame

[
Decrease the length of the normals of the current frame by 1 pixel.
]
Increase the length of the normals of the current frame by 1 pixel.

{
Decrease the distance of the cytosol contour from the membrane contour by 1 pixel.
}
Increase the distance of the cytosol contour from the membrane contour by 1 pixel.

=
Decrease the distance between the equidistant points along the membrane contour by 1 pixel.
-
Increase the distance between the equidistant points along the membrane contour by 1 pixel.

enter
Automatically draw a membrane contour, based on the threshold.

←
Go to previous frame
control-**←**
Go to previous frame and draw a copy the background region of the current frame.
alt-**←**
Go to previous frame and draw a copy the cytosol region of the current frame.
shift-**←**
Go to previous frame and automatically draw a membrane contour, based on the threshold.

→
Go to next frame
control-**→**
Go to next frame and draw a copy the background region of the current frame.
alt-**→**
Go to next frame and draw a copy the cytosol region of the current frame.
shift-**→**
Go to next frame and automatically draw a membrane contour, based on the threshold.

↑
Go to the first channel

↓
Go to the second channel

home
go to the first frame

end
go to the last frame
